# Relationships between Maternal Obesity and Maternal and Neonatal Iron Status

**DOI:** 10.3390/nu10081000

**Published:** 2018-07-30

**Authors:** Angela C. Flynn, Shahina Begum, Sara L. White, Kathryn Dalrymple, Carolyn Gill, Nisreen A. Alwan, Mairead Kiely, Gladys Latunde-Dada, Ruth Bell, Annette L. Briley, Scott M. Nelson, Eugene Oteng-Ntim, Jane Sandall, Thomas A. Sanders, Melissa Whitworth, Deirdre M. Murray, Louise C. Kenny, Lucilla Poston, on behalf of the SCOPE and UPBEAT Consortiums

**Affiliations:** 1 Department of Women and Children’s Health, Faculty of Life Sciences and Medicine, King’s College London, 10th Floor North Wing St Thomas’ Hospital, London SE1 7EH, UK; shahinabegum@live.co.uk (S.B.); sara.white@kcl.ac.uk (S.L.W.); kathryn.dalrymple@kcl.ac.uk (K.D.); carolyn.gill@kcl.ac.uk (C.G.); annette.l.briley@kcl.ac.uk (A.L.B.); eugene.oteng-ntim@gstt.nhs.uk (E.O.-N.); jane.sandall@kcl.ac.uk (J.S.); lucilla.poston@kcl.ac.uk (L.P.); 2 Academic Unit of Primary Care and Population Sciences, Faculty of Medicine, University of Southampton, Southampton General Hospital, Tremona Road, Southampton SO16 6YD, UK; n.a.alwan@soton.ac.uk; 3 NIHR Southampton Biomedical Research Centre, University of Southampton and University Hospital Southampton NHS Foundation Trust, Southampton SO16 6YD, UK; 4 School of Food and Nutritional Sciences, Food Science Building, University College Cork, T12 Y337 Cork, Ireland; m.kiely@ucc.ie; 5 The Irish Centre for Fetal and Neonatal Translational Research (INFANT), Department of Obstetrics and Gynaecology, University College Cork, T12 Y337 Cork, Ireland; d.murray@ucc.ie (D.M.M.); Louise.Kenny@liverpool.ac.uk (L.C.K.); 6 Department of Nutritional Sciences, King’s College London, Franklin-Wilkins Building, 150 Stamford Street, London SE1 7EH, UK; yemisi.latunde-dada@kcl.ac.uk (G.L.-D.); tom.sanders@kcl.ac.uk (T.A.S.); 7 Institute of Health & Society, Baddiley-Clark Building, Richardson Road, Newcastle upon Tyne NE2 4AX, UK; ruth.bell@newcastle.ac.uk; 8 School of Medicine, University of Glasgow, New Lister Building, Glasgow Royal Infirmary, Glasgow G31 2ER, UK; scott.nelson@glasgow.ac.uk; 9 Manchester University NHS Foundation Trust, St Mary’s Hospital, Manchester M13 9WL, UK; melissa.whitworth@cmft.nhs.uk; 10 Department of Paediatrics & Child Health, University College Cork, T12 Y337 Cork, Ireland; 11 Department of Obstetrics and Gynaecology, University College Cork, T12 Y337 Cork, Ireland

**Keywords:** pregnancy, obesity, inflammation, iron status

## Abstract

Obesity in pregnancy may negatively influence maternal and infant iron status. The aim of this study was to examine the association of obesity with inflammatory and iron status in both mother and infant in two prospective studies in pregnancy: UPBEAT and SCOPE. Maternal blood samples from obese (*n* = 245, BMI ≥ 30 kg/m^2^) and normal weight (*n* = 245, BMI < 25 kg/m^2^) age matched pregnant women collected at approximately 15 weeks’ gestation, and umbilical cord blood samples collected at delivery, were analysed for a range of inflammatory and iron status biomarkers. Concentrations of C- reactive protein and Interleukin-6 in obese women compared to normal weight women were indicative of an inflammatory response. Soluble transferrin receptor (sTfR) concentration [18.37 nmol/L (SD 5.65) vs. 13.15 nmol/L (SD 2.33)] and the ratio of sTfR and serum ferritin [1.03 (SD 0.56) vs. 0.69 (SD 0.23)] were significantly higher in obese women compared to normal weight women (*P* < 0.001). Women from ethnic minority groups (*n* = 64) had higher sTfR concentration compared with white women. There was no difference in maternal hepcidin between obese and normal weight women. Iron status determined by cord ferritin was not statistically different in neonates born to obese women compared with neonates born to normal weight women when adjusted for potential confounding variables. Obesity is negatively associated with markers of maternal iron status, with ethnic minority women having poorer iron statuses than white women.

## 1. Introduction

Normal fetal growth and development is dependent upon maternal iron sufficiency during pregnancy. Pregnant women have an increased requirement of iron to support fetoplacental development, expansion of maternal red blood cell mass, and to compensate for intrapartum blood loss; to meet this need, absorption of dietary iron is enhanced concomitant with increased utilisation of existing iron stores [1]. Iron deficiency (ID) is common in young women of reproductive age; in Europe, a recent estimate suggests that the prevalence of ID is 10 to 32% [2]. Maternal ID is a major cause of maternal morbidity [3], and is linked to a higher risk of preterm delivery, low birthweight, and adverse effects on infant neurodevelopment [4,5,6,7,8]. In infancy, ID has long-term effects on cognitive function [9].

Rates of obesity in pregnancy have significantly increased in recent years, with UK women reported to have the highest prevalence in Europe (25.2%) [10], and maternal obesity is associated with most major adverse maternal and fetal outcomes [11]. In non-pregnant populations, there is evidence to suggest an association between obesity and increased risk of ID, with a meta-analysis of the available data concluding that overweight and obese individuals have lower serum iron, transferrin saturation, and a higher risk of iron deficiency [12].

The link between obesity and ID may be attributable to adiposity-related inflammatory mediators on iron regulatory pathways. The proinflammatory cytokine interleukin-6 (IL-6), frequently elevated in obesity, has been shown to induce expression of hepcidin [13], a negative regulator of intestinal iron absorption, macrophage iron efflux and mobilisation of hepatic iron stores [14]. In non-pregnant obese women, hepcidin is upregulated [15]. During a healthy pregnancy, hepcidin is reduced, enabling increased iron transfer to the fetus [1]. It follows that obesity in pregnancy may lead to hepcidin excess and decreased iron transfer to the fetus. The relationship between maternal obesity, inflammation, and iron status has not been extensively examined, with recent conflicting results [16,17,18,19,20]. Studies to date have been limited by small sample sizes and a lack of consensus regarding the measurement of iron and inflammatory biomarkers. There is also a paucity of studies examining the influence of maternal obesity on neonatal iron status, with some indicating that iron status may be compromised [16,17,21,22,23], and others reporting no impact [18].

In this study, we aimed to clarify the relationship between maternal adiposity, inflammation, and iron status in both mother and infant in cohort studies with matched controls comparing obese with normal weight women, drawn from two available prospective studies in pregnancy: the UK Pregnancies Better Eating and Activity Trial (UPBEAT) [24] and the Screening for Pregnancy Endpoints (SCOPE) [25] study. We hypothesized that maternal obesity negatively impacts upon maternal and neonatal iron status via obesity-driven inflammation.

## 2. Methods

### 2.1. Study Design and Participants

To assess the association of obesity with maternal and neonatal iron status, participants from two cohort studies with matched controls were included. Obese (body mass index (BMI) ≥ 30 kg/m^2^) and normal weight (BMI < 25 kg/m^2^) pregnant women, and their neonates, from the UPBEAT and the SCOPE studies were included. Women were matched for age. A sample size of 245 women in each group (obese and normal weight groups) was estimated to provide 96.4% power to detect a 20% difference in serum hepcidin levels, between obese and normal weight controls.

UPBEAT was a multicenter, randomised controlled trial which evaluated the effect of a behavioural intervention of dietary and physical activity advice to improve clinical outcomes in 1555 obese pregnant women [24]. UPBEAT primarily focused on preventing gestational diabetes (GDM) and reducing the incidence of associated large-for-gestational age (LGA) infants. We have previously reported that the intervention was not effective in the prevention of maternal GDM or LGA infants. In UPBEAT, women with a BMI ≥ 30 kg/m^2^ and a singleton pregnancy between 15^+0^ and 18^+6^ weeks’ gestation were recruited across eight study centres in the UK. Exclusion criteria included women with pre-existing diseases, or those who were unable or unwilling to give written informed consent. The current study included data from 245 UPBEAT participants (BMI ≥ 30 kg/m^2^) and their infants from the Guys and St. Thomas’ NHS Trust centre who provided matched maternal blood samples (15^+0^–18^+6^ weeks’ gestation) and cord blood samples.

The SCOPE study aimed to examine early indictors of pregnancy complications as part of an international multicentre study [25]. In SCOPE, healthy, nulliparous women with a singleton pregnancy were recruited before 15 weeks’ gestation. Exclusion criteria included women with an increased risk of pre-eclampsia, small for gestational age (SGA) infants, or preterm birth due to underlying medical conditions. Data from 245 normal weight women (BMI < 25 kg/m^2^) and their infants who participated in SCOPE in Cork, Ireland (*n* = 1768) and the associated BASELINE Birth Cohort Study [26] who provided matched maternal and infant blood samples were included in the present study. In both cohorts, following matching for age, women and their infants for which both samples were available were included by sequential date of recruitment to avoid selection bias.

Both the UPBEAT and SCOPE studies were conducted in accordance with the Declaration of Helsinki. Ethical approval for UPBEAT was granted by the NHS Research Ethics Committee (UK IRAS, Integrated Research Application System; reference 09/H0802/5) and for SCOPE by the Clinical Research Ethics Committee of the Cork teaching hospitals (SCOPE: ECM5(10)05/02/2008).

### 2.2. Data and Sample Collection

At 15 weeks’ gestation in SCOPE, and between 15^+0^–18^+6^ weeks’ gestation in UPBEAT, sociodemographic and anthropometric measurements, including objectively measured weight, were collected. For UPBEAT participants, Class I obesity was defined as 30.0–34.9 kg/m^2^, Class II as 35.0–39.9 kg/m^2^ and Class III as ≥40 kg/m^2^ [27]. In UPBEAT, maternal blood samples were collected and stored at −80 °C, and in the infants, cord blood was collected following delivery [24]. In SCOPE, cord blood was collected as part of the BASELINE birth cohort study, as previously described [26]. Information on maternal and neonatal outcomes was collected throughout pregnancy and at delivery.

### 2.3. Biochemical Measurements

Ferritin (ADVIA Centaur^®^ Ferritin assay, ferritin reagent supplied by Siemens Healthcare Diagnostics Ltd., Surrey, UK), soluble transferrin receptor (No. DTFR1, R & D Systems, Abington, UK), serum hepcidin (EIA5782, DRG Instruments GmbH, Marburg, Germany), UPBEAT IL-6 (No. HS600B, R & D Systems Abingdon, UK), SCOPE IL-6 (No. D6050, R & D Systems, Abingdon, UK) were assessed using standardized enzyme-linked immunosorbent assays (ELISAs) as per the manufacturer’s instructions. C-reactive protein (CRP) was measured using Immunoturbidimetric Assay (Randox Laboratories Ltd., Antrim, UK).

Intra assay coefficients of variation were less than 3% for ferritin, 7.1% for soluble transferrin receptor, 5.68% for hepcidin, 4.2% for IL-6 (SCOPE), 7.8% for IL-6 (UPBEAT), and 2.66% for CRP. Inter assay coefficients of variation were less than 5.4% for ferritin, 6.4% for soluble transferrin receptor, 14.35% for hepcidin, 6.4% for IL-6 (SCOPE), 9.6% for IL-6 (UPBEAT), and 4.44% for CRP.

The World Health Organisation’s (WHO) cut off of 15 µg/L was used to indicate depleted maternal iron stores [28] and cord ferritin concentration <76 µg/L indicated low iron stores at birth [29]. Maternal iron status was principally assessed using the ratio of soluble transferrin receptor (sTfR) and serum ferritin (sF). sTfR values were converted from nmol/L to µg/L by multiplying by 0.075. The sTfR-sF ratio was calculated by dividing sTfR by the log_10_ of sF [20,30]. For the best method of comparison, elevated CRP was indicated by a cut off of CRP > 5 mg/L and elevated IL-6 by a cut off of IL-6 > 1 pg/mL.

### 2.4. Statistical Analysis

The normality of biochemical variables were investigated using distributional plots. Descriptive statistics are presented as mean (standard deviation), frequency (percentage), or median (interquartile range), as appropriate. Bivariate correlations were analyzed by Pearson or Spearman’s correlation. For comparisons between obese and normal weight women, univariate analysis was performed using Independent samples *t* test, analysis of variance (ANOVA) or Mann-Whitney *U* test for continuous variables. For categorical variables, chi-squared test or Fisher’s exact test were used as appropriate.

To assess the association of maternal BMI with maternal inflammatory and iron status, multiple linear and logistic regression was performed on outcomes. The models were adjusted for ethnicity, parity, educational level and smoking. To examine the association of maternal obesity with neonatal iron status, multiple linear and logistic regression was used, adjusted for ethnicity, parity, maternal smoking, gestational age at delivery, and caesarean section birth. Sensitivity analyses were performed excluding women from ethnic minority groups and comparing maternal and neonatal data in white women only.

Statistical analysis was carried out using Stata version 15 (StataCorp, College Station, TX, USA).

## 3. Results

Four hundred and ninety women—245 obese and 245 normal weight controls—were included. The characteristics of the study populations are shown in Table 1.

The median BMI of the obese women was 34.7 kg/m^2^ (interquartile range 32.8–38.0), and the normal weight women 22.4 kg/m^2^ (interquartile range 21.0–23.6). Among obese pregnant women, by WHO obesity classification, 131 (53.5%) had Class I obesity, 70 (28.6%) had Class II obesity and 44 (18.0%) had Class III obesity.

All normal weight women were of white ethnicity, while obese women were predominantly of white ethnicity (73.9%), with the remainder from black (15.5%) or other ethnic groups (10.6%), (*P* < 0.001). Nearly half of obese women were nulliparous (49.8%), compared to all normal weight women (*P* < 0.001). Educational level was lower in obese women (*P* = 0.002), and a higher percentage of obese women had a caesarean section compared to the normal weight women (37.6% vs. 24.5%, *P* = 0.002).

All infant characteristics were similar between obese and normal weight women, apart from gestational age at delivery, which was greater in normal weight women (*P* = 0.01).

### 3.1. Maternal Inflammatory and Iron Status

Obese women had elevated C-reactive protein (CRP > 5 mg/L), compared with normal weight women (66.94% vs. 28.57%, *P* < 0.001), and 96% obese women had an IL-6 concentration above 1 pg/mL, compared with 53% normal weight women (*P* < 0.001) (Table 2 and Supplementary Table S1).

There was no difference in serum ferritin between obese and normal weight pregnant women. In univariate analysis, there was a significant difference in the proportion of women with iron depletion according to the WHO cut-off of 15 µg/L; however, this relationship was attenuated to the null in the adjusted model.

In the adjusted model, mean sTfR concentration was significantly higher in obese women compared to normal weight women [18.37 nmol/L (SD 5.65)] vs. 13.15 nmol/L (SD 2.33)], *P* < 0.001). In a stratified analysis, black women had the highest sTfR concentration [(black 21.89 nmol/L (SD 6.69), white 14.97 nmol/L (SD 4.22), other ethnic group 19.75 nmol/L (SD 6.80)]. Using the ratio of sTfR and serum ferritin, obese women [1.03 (SD 0.56)] had significantly higher levels compared with normal weight women [0.69 (SD 0.23), *P* < 0.001].

The sensitivity analysis including white women only showed the association of maternal BMI with maternal inflammatory, and iron status remained significant after adjustment for confounders for CRP, IL-6, sTfR and the ratio of sTfR (*P* < 0.001).

There was no difference in serum hepcidin concentration between obese and normal weight women. Within the obese group, there was a weak positive correlation between BMI and serum hepcidin which was statistically significant, *r*_s_ = 0.15, *P* = 0.02. Categorising by obesity class showed that serum hepcidin was higher in women with Class II and III obesity, [Class I 8.08 ng/mL (SD 8.42), Class II 9.56 ng/mL (SD 8.19), Class III 10.45 ng/mL (SD 10.09)]; however, there was no significant difference between women classified as obese and their normal weight counterparts (ANOVA, *P* = 0.3).

### 3.2. Neonatal Inflammatory and Iron Status

In order to investigate the association of maternal obesity with neonatal iron status, we examined inflammatory and iron metabolism markers in cord blood obtained at delivery. In univariate analysis, median ferritin was lower in cord blood from neonates born to obese women, and more neonates born to obese women had depleted iron stores (Ferritin < 76 μg/L) [29] compared with neonates born to normal weight women (Table 3 and Supplementary Table S2). Both relationships were attenuated in the adjusted model. Further dividing ferritin by ethnic group, infants born to black women had the lowest ferritin compared with other ethnic groups (black 86.1 μg/L (interquartile range 55.7–146.6), white 154.95 μg/L (interquartile range 105.2–226.6), other ethnic group 96.1 μg/L (interquartile range 60.8–152.1)).

## 4. Discussion

In this nested cohort comparison of two prospective pregnancy studies, obesity was negatively associated with maternal iron status, as evidenced by measurement of soluble transferrin receptor and the soluble transferrin receptor to serum ferritin ratio. There was no difference, however, in serum hepcidin between obese and normal weight pregnant women. Furthermore, we report an association between ethnicity and maternal and infant iron status in obese pregnancies, with the observation that obese pregnant women from ethnic minority groups and their infants had poorer iron status compared to white women.

Relatively few studies have examined the association of maternal BMI with iron status. The current study showing that obesity adversely affected iron biomarkers of pregnant women adds to the growing evidence suggesting that maternal obesity negatively impacts maternal iron status [17,19,20]. In a longitudinal study of 240 pregnant women of heterogeneous BMI, Garcia-Valdes et al. found no effect of BMI on iron status in mid-gestation (24 weeks’ gestation); however, they reported lower sF concentration in obese women compared with normal weight women at term [20]. In contrast, in a longitudinal analysis of Chinese women, Jones et al., reported higher sTfR in obese women at mid-gestation (approximately 20 weeks’ gestation) compared with normal weight women, though the increase in sTfR from mid to late gestation was less pronounced among obese women [17]. Although the sample size in Jones et al. was large (*n* = 1084), only 77 women were classified as obese. In 23 obese and 25 normal weight women, Flores-Quijano et al., showed that sTfR concentration increased more in the obese group over the gestational period [19]. The current study confirms previous observations and reports that the association of obesity with maternal iron status is evident from as early as 15–18 weeks’ gestation.

Moreover, the outcome that poorer maternal iron status in obese ethnic minority women was evident compared to white women adds to the growing evidence of ethnic differences in maternal iron status. The association between iron status and ethnicity has been reported in non-pregnant female populations. Recent data from the US National Health and Nutrition Examination Survey showed that Mexican American and non-Hispanic black women had a greater risk of iron deficiency compared with non-Hispanic white women [31]. Few studies have investigated the association of ethnicity with maternal iron status. The ethnically-diverse HEIRS Study, from the US and Canada, examined the prevalence and predictors of iron deficiency and elevated iron stores in women of reproductive age, including pregnant women. African American and Hispanic ethnicities were associated with iron deficiency (sF concentration ≤ 15 mg/L) in non-pregnant women, whereas the opposite effect was seen in pregnancy, where African American ethnicity was associated with a decreased risk of iron deficiency, and increased iron stores [32]. Although we did not assess dietary iron intake and supplements in the current study, it is plausible that disparity in nutrient intakes exists between white and ethnic minority women. Previous studies have demonstrated that iron intake in pregnancy is inadequate in ethnic minority women [33,34]. As ethnic minority groups experience higher rates of obesity, further studies exploring ethnic disparities in markers of maternal iron status are warranted.

Previous reports suggest a negative impact of maternal BMI on neonatal iron status [16,17,22,23]. Dao et al. showed that serum iron and transferrin saturation in cord blood were lower in neonates born to obese women compared with those of a normal weight [16], and in 316 newborns, Philips et al. found that compared to non-obese pregnant women (BMI < 30 kg/m^2^), obese women delivered offspring with poorer iron status, as assessed using sF and zinc protoporphyrin/heme [23]. In an analysis of the SCOPE-BASELINE pregnancy and birth cohort study, McCarthy et al. showed that maternal obesity at 15 weeks of gestation was inversely associated with cord serum ferritin concentrations [21]. In the present study, poorer iron status determined by cord ferritin were evident in the neonates of obese women, compared with those born to normal weight women. However, this did not achieve significance in the adjusted model, and appeared to be driven by ethnicity, with neonates born to ethnic minority women having poorer iron stores compared with white women. To the best of our knowledge, the association between obesity and ethnicity on neonatal iron status has not been reported previously. Taken together, the findings suggest that among ethnic minority women, iron availability is less, and placental iron transfer is compromised. It is plausible that ethnic dissimilarities in fetal hepcidin could explain the finding that poorer iron status was apparent in infants of ethnic minority women, as fetal hepcidin is implicated in placental iron transfer [1].

Several hypotheses have been proposed to explain the link between obesity and impaired iron status, including poor dietary intake, increased plasma volume, or impaired iron absorption in obese individuals [35]. In non-pregnant populations the low grade inflammation of obesity is associated with increased hepcidin, leading to iron sequestration, and decreased circulating iron [15]. Although concentrations of CRP and IL-6 were greater in obese compared with normal weight pregnant women in this study is consistent with previous reports [16,18,19], serum hepcidin was comparable between the obese and normal weight women. In an exploratory analysis, we found no relationship between CRP or Il-6 and hepcidin in either obese or normal weight women (data not shown). This might indicate that the association between inflammatory mediators and hepcidin is not extant in pregnancy, and that other factors may play an important regulatory role.

Serum hepcidin was elevated in Class II and Class III obese pregnant women compared with the normal weight women in this study. This accords with the evidence of a study in pregnant adolescents, in which a higher grade of obesity was associated with increased hepcidin [18]. The authors conclusion that a high degree of obesity may be necessary to elicit a significant increase in hepcidin [18] may explain the lack of hepcidin increase in the current study, in which the majority of obese women were Class I obese. Hence, there are possibly other regulators, rather than hepcidin, modulating ID in obese pregnant subjects in the current study.

The strengths and weaknesses of the current study warrant consideration. We examined iron status in early pregnancy, i.e., before plasma volume expansion occurs, which can lead to problems in interpretation [36]. A broad range of iron biomarkers were assessed in the present study. This included the measurement of maternal sTfR and the sTfR:sF ratio, which are not affected by the acute-phase response. Furthermore, the assessment of inflammatory markers and the iron regulatory hormone hepcidin provided the opportunity to examine the mechanism linking maternal adiposity to iron deficiency.

Limitations of the study included the fact that the data was derived from two different studies which differed in maternal characteristics including ethnicity and parity. Additionally, the groups were not matched for ethnicity. There was a lack of information on dietary iron intake and iron supplementation, which prevented adjustment for the confounding effect of iron intake on the relationship between obesity and iron deficiency in pregnancy. Markers of iron status were not measured in late gestation when iron transfer to the fetus may be more evident. We measured ferritin in cord blood, which may have been less sensitive to measurements of the effects of a disturbance in iron homeostasis. Moreover, hepcidin levels in cord blood, which could have provided further insight into how maternal obesity can affect placental iron transfer were not measured. Information on GDM was missing in the SCOPE cohort, as, being a low risk population, the oral glucose tolerance test for diagnosis of GDM was not routinely performed.

## 5. Conclusions

As the prevalence of obesity in pregnancy continues to increase, the independent consequences of maternal iron deficiency and maternal obesity have been well described and have significant public health implications. Our findings add to the growing evidence of the detrimental effect of obesity on maternal iron status, which may be an additional risk factor, but hepcidin was not a regulatory factor. Although previous data provides support for a model in which inflammatory-driven alterations in hepcidin result in iron deficiency during obesity, we found no evidence to suggest this, despite demonstration with two markers of an inflammatory status in obese women. Taken together, this study reports, for first time to our knowledge, the negative influence of obesity and ethnicity on maternal and infant iron status. Further research is warranted to investigate the effect of obesity and ethnicity on disturbances to iron metabolism in both mothers and infants.

## Figures and Tables

**Table 1:** Maternal and infant characteristics of obese (*n* = 245) and normal weight (*n* = 245) pregnant women.

	Obese (*n* = 245)	Normal Weight (*n* = 245)	*P* ^#^
**Maternal characteristics**			
**Age (years)**	30 (4.2)	30 (4.2)	1.00
**BMI (kg/m^2^)**	34.7 (32.8–38)	22.4 (21–23.6)	<0.001
**Ethnicity**			<0.001
Black	38 (15.5)	0	
White	181 (73.9)	245 (100)	
Other ethnic group	26 (10.6)	0	
**Booking haemoglobin**	127.5 (9.9)	128.1 (8.2)	0.4
**Parity**			<0.001
Nulliparous	122 (49.8)	245 (100)	
**Smoking**			0.07
Smoking at 1st visit	21 (8.6)	14 (5.7)	
Ex-smoker	59 (24.1)	43 (17.6)	
Never	165 (67.4)	188 (76.7)	
**Educational level (<12 years)**	27 (11.0)	9 (3.7)	0.002
**Gestational Diabetes**	75 (31.0)	- *	
**Pre-eclampsia**	9 (3.7)	5 (2.0)	0.3
**Caesarean section**	92 (37.6)	60 (24.5)	0.002
**Infant characteristics**			
Gestational age at delivery	39.7 (38.7–40.9)	40.3 (39.3–41)	0.01
Male	128 (52.2)	128 (52.2)	1.00
Preterm birth < 37 weeks	10 (4.1)	7 (2.9)	0.5
Birthweight (g)	3595 (3240–3855)	3480 (3150–3760)	0.07
Large for gestational age ^+^	22 (9.0)	33 (13.5)	0.1
Small for gestational age	22 (9.0)	17 (6.9)	0.4

Data are presented as mean (SD), median (interquartile range) or number (%). ^#^, Differences between maternal and infant characteristics between groups assessed using Independent samples *t* test, or Mann-Whitney *U* test for continuous variables, and chi-squared test for categorical variables. *, Not routinely tested in all women. ^+^, Customised centile, adjusting for maternal height and weight, ethnic origin, parity, and sex of the baby.

**Table 2:** Maternal inflammatory and iron status indicators of obese and normal weight pregnant women.

Indicator	Obese (*n* = 245)	Normal Weight (*n* = 245)	Unadjusted Analysis *P* Value	Adjusted Analysis *P* Value *^#^*
Ferritin (ug/L)	31.6 (17.9–55.9)	34.2 (20–54.9)	0.2	0.8
Ferritin < 15 ug/L (%)	51 (20.82)	34 (13.88)	0.04	0.7
Soluble transferrin receptor (nmol/L)	18.37 (5.65)	13.15 (2.33)	<0.001	<0.001
sTfR:log_10_sF	1.03 (0.56)	0.69 (0.23)	<0.001	<0.001
Hepcidin (ng/mL)	8.93 (8.69)	8.95 (7.43)	0.97	0.96
CRP > 5mg/L (%)	164 (66.94)	70 (28.57)	<0.001	<0.001
IL-6 > 1 pg/mL (%)	237 (96.73)	130 (53.06)	<0.001	<0.001

Continuous variables presented as mean (SD) or median (interquartile range). Categorical variables presented as n (%). ^#^, Multiple linear or logistic regression, adjusted for ethnicity, parity, educational level and smoking. sF, serum ferritin; sTfR, soluble transferrin receptor; CRP, c-reactive protein; IL-6, interleukin 6.

**Table 3:** Inflammatory and iron status indicators of neonates of obese and normal weight pregnant women.

Indicator	Obese (*n* = 245)	Normal Weight (*n* = 245)	Unadjusted Analysis *P* value	Adjusted Analysis *P* value ^#^
Ferritin (μg/L)	130.7 (80.5–199.1)	162.8 (119.5–226.6)	<0.001	0.08
Ferritin < 76 ug/L (%)	54 (22.04)	29 (11.84)	0.003	0.3
CRP > 5 mg/L (%)	5 (2.04)	0 (0)	0.06	-

Continuous variable presented as median (interquartile range). Categorical variables presented as *n* (%). ^#^, Multiple linear or logistic regression, adjusted for ethnicity, parity, smoking, gestational age at delivery, and caesarean delivery. CRP, c-reactive protein.

## Supplementary Materials

The following are available online at http://www.mdpi.com/2072-6643/10/8/1000/s1, Table S1: Association between maternal obesity and maternal inflammatory and iron status. Differences and 95% confidence intervals, Table S2: Association between maternal obesity and neonatal iron status. Differences and 95% confidence intervals.
