# Supplementary material for: Relationships between Maternal Obesity and Maternal and Neonatal Iron Status
Source: Nutrients. 2018 Jul 30;10(8):1000. doi: 10.3390/nu10081000 (PMC6115715; doi:10.3390/nu10081000)
Supplement: Supplementary file 1 [file nutrients-10-01000-s001.pdf]

Table S1. Association between maternal obesity and maternal inflammatory and iron status. Differences and 95% confidence intervals.

Indicator	BMI category	Adjusted difference	95% CI
Ferritin (ug/l)	Normal weight	Reference	
	Obese	0.03	(-0.15, 0.20)
Ferritin <15ug/l*	Normal weight	Reference	
	Obese	1.13	(0.58, 2.19)
Soluble transferrin receptor (nmol/l)	Normal weight	Reference	
	Obese	3.43	(2.50, 4.35)
sTfR:log_{10}sF	Normal weight	Reference	
	Obese	0.17	(0.08, 0.26)
Hepcidin (ng/ml)	Normal weight	Reference	
	Obese	0.04	(-1.79, 1.88)
CRP>5mg/l*	Normal weight	Reference	
	Obese	0.20	(0.12, 0.33)
IL-6>1pg/ml*	Normal weight	Reference	
	Obese	0.02	(0.00, 0.07)

Multiple linear regression, adjusted for ethnicity, parity, educational level and smoking. *Multiple logistic regression adjusted for ethnicity, parity, educational level and smoking. sF, ferritin; sTfR, soluble transferrin receptor; CRP, c-reactive protein; IL-6, interleukin 6.

Table S2. Association between maternal obesity and neonatal iron status. Differences and 95% confidence intervals.

Indicator	BMI category	Adjusted difference	95% CI
Ferritin (ug/l)	Normal weight	Reference	
	Obese	-0.12	(-0.26, 0.01)
Ferritin <76ug/l*	Normal weight	Reference	
	Obese	0.73	(0.39, 1.37)

Multiple linear or logistic regression, adjusted for ethnicity, parity, smoking, gestational age at delivery, and caesarean delivery. *Multiple logistic regression, adjusted for ethnicity, parity, smoking, gestational age at delivery, and caesarean delivery.
